# Ayurveda for all: 11 action points for 2011

**DOI:** 10.4103/0975-9476.74416

**Published:** 2010

**Authors:** Bhushan Patwardhan

**Affiliations:** *Editor-in-Chief, Institute of Ayurveda and Integrative Medicine, Bangalore, India. E-mail: bpatwardhan@gmail.com*

As the year 2010 drew to an end, we witnessed the Fourth World Ayurveda Congress (WAC) with its central theme ‘Ayurveda for All’. Attracting over 4000 delegates from different parts of India, 300 foreign delegates from over 60 countries and over 3,00,000 visitors to the ‘Arogya expo’ health exhibition, the 4^th^ World Ayurveda Congress has created a new benchmark. A galaxy of eminent vaidyas, scientists, industrialists, policy makers and opinion leaders gave thought provoking plenary talks and keynote, lectures. Over 550 papers and posters were presented by researchers and young students, and several pre- congress workshops and symposia were attended by overwhelming numbers. These 4^th^ WAC figures offer but a glimpse of Ayurveda’s growing popularity, and the increasing public curiosity towards all its aspects. The organizer s, especially Vijanan Bharati, the Department of AYUSH, Government of India, and the Government of Karnataka deserve our thanks and admiration for all that they did to make the Congress the success it was. As its official Journal, J-AIM will soon be publishing a special issue of proceedings and select presentations.

The year 2011 promises to be a very crucial year for the AUS (Ayurveda, Unani, and Siddha) sector, especially as the world is seeking affordable health solutions and western medicine is facing a crisis. Ayurveda is the world’s most comprehensive, personalized, holistic and sustainable health system; it is based on sound philosophical and scientific principles, and is now poised to regain the global leadership it lost in the 19^th^ century. The reasons are many: Ayurveda provides much more than just a system of medicine - it offers the keys to a healthy way of life and living; better understanding of how systemic theories of Ayurveda ‘shastra’ can provide new insights and paradigms in the biomedical ‘sciences’. However, to fulfil this realization several things are urgently needed including priority to human resource development, capacity building and resource optimization. Only then can Ayurveda meet the growing aspirations of students, faculty, practitioners, researchers, industries and, most importantly, millions of health seeking people.

The Department of AYUSH has a vision to reinvigorate the AYUSH systems and promote holistic health. It has a laudable mission to mainstream AYUSH at all levels in the health care system; to improve access to and quality of public health delivery and to focus on promotion of health and prevention of diseases.[1] Former Secretaries, especially Ms Malati Sinha and Ms S. Jalaja, have contributed significantly to strengthen the foundations of the department and have initiated many good schemes, programs and projects. Commendable efforts have been made by the department, however, the outcomes seem relatively insignificant. Recently, the Department of AYUSH has taken the timely step of requesting its founder Secretary, Ms Shailaja Chandra, to undertake a review and gap analysis to know the actual benefits that the AUS Systems bring to the people. Ms Chandra comes with rich experience and good understanding of this sector. It is hoped that her report will be useful during the 12^th^ Plan exercise. It is important that all stakeholders respond to her proactively and offer their experiences, comments and suggestions. Based on interactions with various stakeholders assembled in large numbers for the 4^th^ WAC, we wish to propose an 11 point action agenda for the year 2011 and beyond.

## 1. MAINSTREAMING

AYUSH is a complex system where each word represents unique features bringing diverse expectations. It is important to maintain unity in this diversity so that a consolidated message strengthening to the Indian medical heritage can be achieved. At present, research in the field shows an unplanned diversity that is not resulting in much significant impact. Efforts to mainstream AYUSH systems in national health policy, programs and public health, merit serious attention and massive support. AYUSH systems are capable of providing primary health care to the masses, as they offer safer treatments for difficult to treat conditions, including safer palliative care, better health restoration and promotion, and unique disease prevention.

## 2.CONCEPTUAL CLARITY

Several areas of understanding springing from basic principles of Ayurveda can shape the future of modern science research. In addition to retrospective analysis to demonstrate the importance of Ayurveda, we need concentrated retroactive and proactive efforts to clarify its potentially enormous benefits to global science and advanced research. Most past and present attempts in Ayurveda research revolve around herbal medicines and related activities that are also important and should continue. However, the real value of Ayurveda lies in its basic principles, including its unique concepts of panchamahabhuta, prakriti, guna, rasa, agni, dosha, dhatu, mala, srotas, and its personalized approach to nidan, chikitsa and rasayana. We need to encourage ambitious high-impact projects to bring contemporary relevance and deeper understanding to Ayurveda principles that are of great strategic importance to India.

## 3.INTER GOVERNMENTAL COORDINATION

Certain significant developments have boosted systematic research on various aspects of Ayurveda, and traditional medicine in India. They include the Golden Triangle project jointly managed by CSIR, ICMR and AYUSH; the CSIR’s New Millennium Indian Technology Leadership Initiative (NMITLI), and various schemes initiated by DST and DBT. Under the ‘Science Initiative in Ayurveda’ research, the DST has supported several collaborative projects on science and Ayurveda involving networks of institutions. It is important to establish systems to ensure that such efforts synergise and multiply.

## 4.INTERNATIONAL CONSENSUS

There is a growing demand from policy makers and regulatory authorities to make AYUSH more evidence-based in order to enable wider acceptance. The internationalization of AYUSH needs to be focused on Indian ethos and needs. The Indian Diaspora in many countries, especially from the SAARC region is looking to India for leadership and support. This calls for clarity on strategy, approaches and methodologies to align AYUSH’s core strengths with ongoing international developments. Our policies and strategies are emerging more as responses and reactions to other countries’ requirements. Proactive leadership in research, practice and advocacy emerging from India is far from what is required or expected. International cooperation needs to be reconsidered and pivoted around India’s own needs and priorities.

## 5.RESEARCH METHODOLOGY

It is important to recognize the need for research in AYUSH systems. However it is not appropriate to use methodologies developed for use in biomedicine. Within their own disciplines, AYUSH systems are inherently evidence based. It is therefore important to distinguish between cross-disciplinary evidence that may need to be created, and evidence already available within AYUSH systems. TCM has evolved appropriate methodologies and reporting standards; AYUSH must now make a priority of undertaking similar exercises in collaboration with WHO, CONSORT and other such bodies. The Central Council for Research in Ayurveda and Sidha (CCRAS) and that for Unani should give priority to this exercise.

## 6.CLINICAL PROTOCOLS

Issues related to appropriateness of conventional biomedical and clinical models for evaluating efficacy of systems of traditional medicine remain critical. A holistic, whole systems approach seems better suited to study therapeutic efficacy and pharmacodynamics of traditional medicines. Instead of randomized controlled trials normally used as gold standard in routine biomedical research, strategies of pragmatic or whole system clinical trials may be better suited for Ayurveda - as has previously been well argued. Guidance from WHO, EMEA, OECD, US FDA and such bodies will remain useful, but finally the onus will remain on India herself to develop and implement appropriate study and clinical protocols to create the necessary evidence base. Again, there is lot to learn from TCM experiences.

## 7.DOCUMENTATION

Demands and exports of formulations, raw materials and herbal extracts are growing and need compliance with global quality standards. Data on consumption, safety and efficacy of Ayurvedic medicines is grossly inadequate, while that regarding their in-country consumption is not readily available. Nor is data on evidence supporting the safety and efficacy of Ayurvedic medicines and practices in India easily available. Systematic documentation of history of use over a defined period in a large population should be carried out as a team India effort using pharmaco-epidemiology principles.

## 8.REGULATION, QUALITY AND SAFETY

Various Government agencies (AYUSH, ICMR, FSSAI) have provided guidance related to traditional and herbal products. The US FDA, EMEA and other regulatory agencies have published practical guidelines for botanical drug development. Such botanical guidance recommends use of different types of data for preliminary safety, and also takes into account large quantities of mostly anecdotal human data instead of the well-controlled animal studies and human trials used in an IND. Distinctions between Ayurvedic medicines, herbal medicine, dietary supplements, health foods, and nutraceuticals need to be clearly stated and regulated. AYUSH products must have independent guidance for quality, safety standards, and a well defined evidence base for their efficacy. The newly conceived Ayurveda Pharmacopoeia Commission should play the key role by involving people with the right kind of experience.

## 9.HUMAN RESOURCE DEVELOPMENT

Attitudes and skill sets are so disparate that Shastra and Science remain poles apart. Those in mainstream science may abstain from Ayurveda since they are unable to accept Ayurvedic solutions because they seem so counterintuitive. This is further aggravated by negative propaganda about Ayurveda. Resolving this dilemma may be a herculean task - like converting the dirt road in a village into an airport landing strip. Achieving this transition will require a strong team of young, enthusiastic and dedicated teachers, who are very strong in both Ayurveda Shastra and science. Such Vaidya Scientists will catalyze the anticipated change, and accelerate the speed of the transition. We need to create world class institutions of excellence like IITs and IIMs to provide capacity building, human resource development and thought leadership to the Ayurveda sector, which is so precious to India.[2]

## 10. LEADERSHIP

The world is a facing major challenge in the health care sector. AYUSH can certainly offer leadership by providing safer, more affordable solutions. The increased visibility and global acceptance of Ayurveda will be enhanced once its rightful leadership position is attained through scientific research that results in quality publications in high impact journals. We need to strengthen existing centers of excellence and create new ones. We need an exemplary institution as an epitome of quality and rigor balancing science with shastra. We hope that key scientific advances will emerge through such efforts giving India and Ayurveda due visibility, acceptance and the much desired boost in the global scientific and industrial fraternity.

## 11.AYURVEDA FOR ALL

This is an ‘Innovation Decade’ and thought leaders are keen to use innovation to benefit the masses. Ayurveda has a clear role to play. The Department of AYUSH must facilitate innovative solutions for affordable public health, promotional and preventive healthcare, and equally to contribute better and safer treatments for chronic, difficult to treat, metabolic and degenerative disorders.

We at J-AIM hope that the Department of AYUSH, the Planning Commission and other Indian Government bodies will introduce a new, visionary charter in the best interests of the people, and lead Ayurveda to assume its much deserved global leadership, shaping the future of medical practice and the health sciences, more widely. J-AIM will remain committed to the revitalization of the Indian medical heritage and to facilitate the desired renaissance for Ayurveda.[3] On this note, on behalf of the Editorial Board, I am happy to present the fourth issue of J-AIM and wish our readers, reviewers and well-wishers all the best for a very happy and healthy New Year, 2011.
